# Dual-Mode Ce-MOF Nanozymes for Rapid and Selective Detection of Hydrogen Sulfide in Aquatic Products

**DOI:** 10.3390/polym16121747

**Published:** 2024-06-20

**Authors:** Qi Cheng, Xiaoyu Du, Zuyao Fu, Zhaoyang Ding, Jing Xie

**Affiliations:** 1 College of Food Science and Technology, Shanghai Ocean University, Shanghai 201306, China; 2131112@st.shou.edu.cn (Q.C.); m220351050@st.shou.edu.cn (X.D.); m230300906@st.shou.edu.cn (Z.F.); 2 Marine Biomedical Science and Technology Innovation Platform of Lin-Gang Special Area, Shanghai 201306, China; 3 Shanghai Engineering Research Center of Aquatic-Product Processing & Preservation, Shanghai 201306, China

**Keywords:** H_2_S, nanozyme, metal–organic frameworks, dual-mode sensor, food freshness

## Abstract

Increasing concern over the safety of consumable products, particularly aquatic products, due to freshness issues, has become a pressing issue. Therefore, ensuring the quality and safety of aquatic products is paramount. To address this, a dual-mode colorimetric–fluorescence sensor utilizing Ce-MOF as a mimic peroxidase to detect H_2_S was developed. Ce-MOF was prepared by a conventional solvothermal synthesis method. Ce-MOF catalyzed the oxidation of 3,3’,5,5’-tetramethylbenzidine (TMB) by hydrogen peroxide (H_2_O_2_) to produce blue oxidized TMB (oxTMB). When dissolved, hydrogen sulfide (H_2_S) was present in the solution, and it inhibited the catalytic effect of Ce-MOF and caused the color of the solution to fade from blue to colorless. This change provided an intuitive indication for the detection of H_2_S. Through steady-state dynamic analysis, the working mechanism of this sensor was elucidated. The sensor exhibited pronounced color changes from blue to colorless, accompanied by a shift in fluorescence from none to light blue. Additionally, UV–vis absorption demonstrated a linear correlation with the H_2_S concentration, ranging from 200 to 2300 µM, with high sensitivity (limit of detection, LOD = 0.262 μM). Fluorescence intensity also showed a linear correlation, ranging from 16 to 320 µM, with high selectivity and sensitivity (LOD = 0.156 μM). These results underscore the sensor’s effectiveness in detecting H_2_S. Furthermore, the sensor enhanced the accuracy of H_2_S detection and fulfilled the requirements for assessing food freshness and safety.

## 1. Introduction

Hydrogen sulfide (H_2_S) is a colorless, corrosive, flammable and toxic gas under standard conditions, characterized by a distinct rotten egg odor at low concentrations. Although it has traditionally been recognized as a key indicator of food spoilage, recent studies have revealed its significant role as a small redox-active molecule in cell signaling pathways [1]. However, H_2_S gas generated during food manufacturing processes can adversely affect food quality. For instance, H_2_S produced by yeast during winemaking can compromise the taste and flavor of wine [2]. Numerous studies have shown that long-term exposure to low doses of H_2_S is associated with headaches, vertigo, respiratory disorders and other related symptoms, and it may cause respiratory diseases. When ingested through food, H_2_S penetrates cell membranes, disrupts the respiratory chain and interferes with cellular respiration. Concurrently, H_2_S, recognized as a physiologically active gas, participates in various physiological processes within the body, including the facilitation of vascular smooth muscle relaxation and the modulation of nerve transmission [3]. Excess H_2_S concentrations can lead to respiratory system paralysis, impairing normal cognitive functions and behaviors. Abnormal H_2_S levels are closely associated with symptoms of various diseases, including acute infectious diseases, food poisoning and severe conditions like Down syndrome, diabetes and Alzheimer’s disease [4], sometimes resulting in sudden death. Given the health risks associated with H_2_S exposure, detecting its presence has become a matter of significant concern.

H_2_S is now recognized as a third gas transmitter alongside nitric oxide (NO) and carbon monoxide (CO) [5]. The detection of gases produced during food manufacturing helps standardize processes and quality control, and it also helps with continuously monitoring their status and preservation and tracing their origin. Establishing a fluorescence-based detection method for the quantitative and visual assessment of H_2_S in aquatic products holds significant practical implications for ensuring food safety and human health.

Traditional analytical techniques include gas chromatography (GC), electrochemical analysis, metal-induced sulfide precipitation [6], plasma atomic emission [7], surface-enhanced Raman scattering [8] and airfield effect transistors [9]. However, these methods necessitate skilled operators and employ complex, costly and stationary equipment [10]. In contrast, the fluorescence method offers higher sensitivity and convenience. Moreover, colorimetric sensors also provide a user-friendly and easy-to-deploy solution for routine food monitoring, as they are easily understood by consumers and do not require sophisticated instrumentation. In addition, their portability and cost-effectiveness make them ideal for widespread adoption in food safety protocols. In summary, the combination of colorimetric and fluorescence methods can achieve multiple advantages, such as portability, sensitivity and a low cost [11].

Colorimetry has garnered significant attention in bioanalysis and environmental science due to its affordability, simplicity and visual monitoring capabilities. To facilitate the translation of detection signals into perceptible color alterations, a variety of compounds and materials have been engineered and synthesized as colorimetric indicators. In the realm of H_2_S sensing, certain colorimetric approaches have been developed, leveraging the catalytic properties of metal–organic frameworks (MOFs) or similar catalysts that oxidize specific substrates in response to H_2_S. The reaction products then interact with color-developing agents, leading to a visible color change that is correlated with H_2_S concentration. This strategy allows for a direct and intuitive assessment of H_2_S levels, which is valuable for environmental and medical monitoring applications.

However, natural peroxidases exhibit high catalytic activity but are susceptible to environmental conditions such as pH and temperature, which can lead to decreased activity or complete inactivation [12]. Consequently, natural peroxidases are impractical for use outside laboratory settings. To enhance the applicability of colorimetric detection, nanomaterials have emerged as promising alternatives to natural peroxidases. Nanomaterial-based enzymatic mimics, or nanozymes, offer several advantages, including simple and cost-effective synthesis, high structural and chemical stability, ease of storage and compatibility with a wide range of operating temperatures and pH values, rendering them more suitable for field-based determinations.

Enzymes, as a class of biological macromolecular catalysts, play pivotal roles in organismal biotransformation and industrial processes. Their notable advantages, such as high efficiency, exceptional selectivity and environmental friendliness, render them indispensable tools across various sectors of modern industry. However, the inherent limitations of natural enzymes—including sensitivity to environmental factors, poor operational stability, high costs and short lifespans—have restricted their broad application, particularly in challenging conditions. Consequently, substantial efforts have been devoted over the past few decades to surmounting these constraints. Prominent strategies encompass directed evolution, protein and enzyme engineering, enzyme immobilization, artificial enzyme design and medium regulation. With dynamic advancements in modern nanomaterials science, supramolecular chemistry and protein engineering, artificial enzyme engineering has emerged as a promising avenue to address these challenges. Artificial enzyme nanomaterials, exemplified by nanozymes, are gaining traction as effective alternatives to natural enzymes due to their controllable catalytic activity, adaptable design, heightened stability and favorable biocompatibility. As a result, they offer compelling prospects for diverse industrial applications [13].

Nanozymes, defined as nanomaterials with natural enzyme-like properties, have gained recognition as promising artificial enzymes in recent years. These nanozymes effectively merge the benefits of traditional chemical catalysts and biocatalysts, exhibiting high and tunable catalytic efficiency, exceptional stability, recyclability, ease of manufacture and cost-effectiveness. Consequently, both the fundamental research and practical applications of nanozymes are flourishing, demonstrating significant potential in areas such as biosensing, environmental resource protection, food safety and antimicrobial and antioxidant applications [14]. Metal–organic frameworks (MOFs), characterized by their porous and crystalline structure, consist of a periodic network formed by the self-assembly of organic units (typically organic ligands such as carboxylic acids and pyridines) and metal ions or clusters (commonly transition metals like Cu^2+^, Zn^2+^ and Fe^2+^). Due to their abundant active sites, customizable properties and robust stability, MOFs are considered promising candidates for nanozyme applications [15,16,17,18].

Additionally, MOFs feature large specific surface areas, adjustable pore sizes and shapes, easy modification and numerous unsaturated metal sites, which make them highly suitable for applications in gas adsorption separation, catalysis, sensing, biomedicine and more [19].

In recent years, a variety of MOF-based nanozymes from the oxidoreductase (such as peroxidase, oxidase, catalase and superoxide dismutase) or hydrolase (such as carbonic anhydrase, phosphatase, protease and nuclease) families have been developed and have achieved significant success in analytical chemistry, environmental management, disease diagnosis and treatment. The outstanding catalytic activity of these nanozymes has also led to their widespread use in constructing colorimetric biosensors, transforming them into powerful and effective tools for real-time detection. Innovative colorimetric sensing strategies have been developed by integrating nanozymes with colorimetric sensors [20]. Furthermore, various nanomaterials, including carbon-based nanomaterials, metal nanoparticles, metal oxide nanoparticles and quantum dots, have been employed for real-time, miniaturized and highly selective H_2_S detection [21]. MOF-based techniques for detecting H_2_S through fluorescence, catalytic and electrochemical properties have been extensively explored, contributing to advancements in the field [22].

**Scheme 1:**
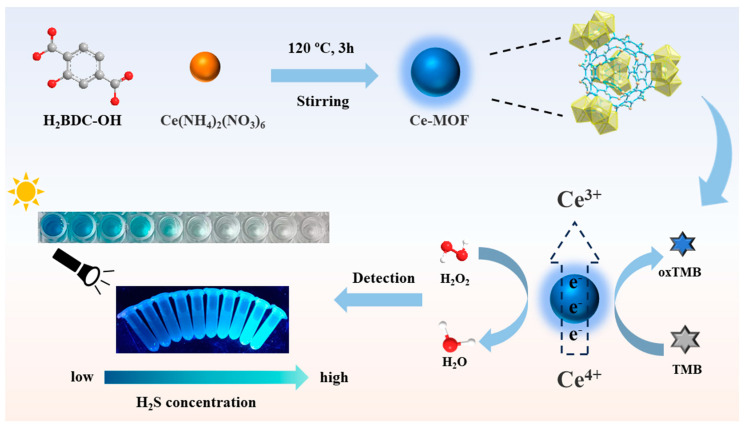
Schematic representation of the preparation of Ce-MOF and the colorimetric–fluorescent sensing of H_2_S.

In this study, a dual-mode colorimetric–fluorescence sensor based on a Ce-MOF nanozyme was developed. The fluorescence resonance energy transfer (FRET) between Ce-MOF and TMB resulted in the generation of bright blue fluorescence in the solution. Furthermore, as the concentration of Na_2_S increased, oxTMB in the solution was reduced back to TMB, resulting in a decrease in oxTMB and a corresponding increase in fluorescence emission intensity within the detection system. This indicated an enhanced FRET effect. For H_2_S detection (Scheme 1), visual fluorescence detection of H_2_S was achieved. Ce-MOF was synthesized using solvothermal synthesis, demonstrating an excellent dual-mode colorimetric fluorescence response. Therefore, the FRET-based Ce-MOF provided a highly selective and sensitive fluorescence detection strategy for H_2_S analysis. The prepared Ce-MOF exhibited exceptional stability and selectivity under the given detection conditions while maintaining uniform particle size distribution of the nanozyme. Based on this approach, our study successfully detected H_2_S levels in aquatic products and confirmed its significant potential for application in food freshness assessments.

## 2. Materials and Methods

### 2.1. Materials

2-hydroxy-terephthalic acid (H_2_BDC–OH), TMB, ammonium cerium nitrate, 3,3′,5,5′-tetramethylbenzidine, N,N-dimethylformamide(DMF), acetic acid, anhydrous ethanol, sodium acetate, hydrogen peroxide, sodium sulfide and other substances used for selectivity and anti-interference testing, such as metal ion solutions, were purchased from Aladdin (Shanghai, China) and used as received. Fresh shrimp and salmon were purchased from Lotus Supermarket (Shanghai, China). The buffer solution of acetic acid/sodium acetate (0.2 M) was obtained by mixing 82 mL of acetic acid (0.2 M) and 18 mL of sodium acetate (0.2 M).

### 2.2. Apparatus

#### 2.2.1. Morphology Characterization

The morphologies of Ce-MOF were investigated using a field emission scanning electron microscope (Tescan Mira 3 XH, Tescan, Brno, Czechia) for SEM and SEM–energy dispersive spectroscopy (EDS) analyses. Additionally, high-resolution images were captured with a transmission electron microscope (Talos F200X G2, Thermo Fisher, Waltam, MA, USA) for TEM analysis. The particle size distribution of Ce-MOF was measured using a dynamic light scattering (DLS) system (Malvern Zetasizer Nano ZS, Malvern Instruments Ltd., Westborough, MA, USA). The system was stabilized at 25 °C with deionized water (0.01 mg·mL^–1^) serving as the dispersion medium in a four-sided quartz cuvette. Approximately 2 mg of the powder sample was used for each test. For SEM and EDS analyses, a scanning voltage of 15 kV was employed. Samples were prepared by applying them to the surface of a conductive adhesive using cotton swabs, coating with gold and then examining in the electron microscope chamber. For TEM, a scanning voltage of 200 kV was utilized. Samples were dispersed in ethanol and homogenized via ultrasonication. Subsequently, a droplet of the suspension was placed on a copper mesh carrier and observed in the TEM once dry. Free radicals produced by memetic peroxidase Ce-MOF were tested by electron paramagnetic resonance (EPR) (Bruker, A300, Mannheim, Germany). The fluorescence lifetime was determined with HORIBA’s highly sensitive integrated fluorescence spectrometer (FluoroMax-4, HORIBA, Kyoto, Japan).

#### 2.2.2. XPS Analysis

X-ray photoelectron spectroscopy (XPS) measurements were conducted using the EscaLab 250Xi XPS system (Thermo Fisher Scientific, Horsham, UK). This analysis offered detailed insights into the chemical states and elemental composition of the sample. For the measurements, approximately 10 mg of the sample was used. Key parameters included an operating voltage of 12.5 kV, a filament current of 16 mA and the use of Al Kα radiation as the excitation source. The measurements were performed with a pass energy of 30 eV and step size increments of 0.1 eV.

#### 2.2.3. FT-IR Analysis

The synthesized MOFs were characterized using Fourier transform infrared spectroscopy (FT-IR) with a Nexus 410 FT-IR spectrometer (Thermo Nicolet, Waltham, MA, USA). Spectra were recorded across a scanning range from 400 to 4000 cm^−1^ for samples weighing approximately 10 mg. Analysis of the FT-IR spectra enabled the identification of specific functional groups within the MOF materials by analyzing peak positions along the IR curve.

#### 2.2.4. XRD Analysis

X-ray diffraction (XRD) patterns were acquired using an Ultima IV multipurpose X-ray diffractometer (manufactured in Tokyo, Japan). Powder X-ray diffraction (XRD) patterns were measured on a Bruker Focus D8 diffractometer operating at 40 kV using Cu-Kα X-ray radiation (λ = 1.54056 nm). The instrumental parameters for the XRD analysis comprised a scanning range from 5 to 80 degrees, a scanning speed of 12 degrees per minute and a sample quantity of approximately 50 mg. The obtained data were then analyzed and processed using Jade 4.6.0 software.

#### 2.2.5. Brunauer–Emmett–Teller (BET) Analysis

Nitrogen adsorption–desorption isotherms were measured by the BET technique. Low-pressure gas adsorption studies were performed on the 3Flex Analyzer (Micromeritics Instrument, Norcross, GA, USA), a fully automated microporous gas analyzer, with relative pressures up to 1 atmosphere. The low temperature was controlled using a liquid nitrogen bath at 77 K. The apparent surface area was determined by applying BET and Langmuir models from nitrogen adsorption isotherms collected at 77 K.

#### 2.2.6. Optical Detection

UV–vis absorption spectra were recorded using a U-3900 UV–vis spectrophotometer (Hitachi, Tokyo, Japan), and fluorescence spectra were captured with an F-7000 fluorescence spectrophotometer (Hitachi, Japan). Additionally, short-wave ultraviolet detection of the samples at 365 nm was performed using a handheld UV lamp (Spectroline, Melville, NY, USA).

### 2.3. Synthesis of Ce-MOF

Ce-MOF was synthesized using the classical solvothermal method [23]. First, 164.5 mg of ammonium cerium nitrate was dissolved in 15 mL of DMF, and 109.3 mg of 2-hydroxyterephthalic acid was dissolved in 45 mL of DMF. Both solutions were sonicated until fully dissolved. The solution containing 2-hydroxyterephthalic acid was then transferred to a 100 mL round-bottomed flask and immersed in silicone oil at 120 °C. A magnetic stirrer was used to maintain stirring at 200 rpm. When the solution in the flask reached approximately 120 °C, 600 µL of glacial acetic acid was added, followed by the slow addition of 15 mL of the ammonium cerium nitrate solution at a rate of 1 drop every 3 s. After the complete addition of the ammonium cerium nitrate solution, the opening of the flask was gently covered with tinfoil, and stirring was continued at 200 rpm for 6 min. Subsequently, the magnetic stirrer was turned off, and the temperature was maintained at 120 °C for 3 h to allow for the synthesis and growth of Ce-MOF. Afterward, the heating was turned off, the flask was removed from the silicone oil, and the solution was cooled to room temperature. The solution was then evenly divided into four 50 mL centrifuge tubes, and 15 mL of anhydrous ethanol was added to each tube. After balancing, the tubes were centrifuged at 12,000 rpm at 4 °C for 20 min. Centrifugation was accomplished, the supernatant was removed, and 20 mL of anhydrous ethanol was added to each tube. This washing step was repeated three times to ensure the removal of any impurities. Once the three washes were completed, the washed precipitate was collected and placed in an oven at 75 °C for 24 h until completely dried, yielding the final product.

### 2.4. The Mimetic Peroxidase-like Activity of Ce-MOF Nanozyme

TMB was used as the color developing substrate. Ce-MOF catalyzes TMB to ox-TMB only in the presence of Ce-MOF, TMB and H_2_O_2_, and the solution was obviously blue, which indicated that it had the activity of simulating peroxidase. The UV–vis absorption peak was obtained at 652 nm, and the ionic system of the solution changed. In order to better understand the properties of Ce-MOF intermediates during enzyme conversion in the presence of hydrogen peroxide and TMB, 1.25 mL of Ce-MOF (0.05 mg·mL^–1^), 8 mL of buffer solution and 0.75 mL of H_2_O_2_ (100 mM) were prepared for EPR.

To further analyze its catalytic activity, the same concentration of Ce-MOF was used in acetic acid/sodium acetate buffer solution (pH = 4, 0.2 M). Various concentrations of TMB (1.5–43.26 mM) and H_2_O_2_ (0.01–2 mM) were tested as the kinetic parameters of the substrates. The absorbance of the solution at 652 nm was rapidly recorded following the final addition of each varying concentration of substrate. The maximum reaction rate (V_max_) and the Michaelis constant (K_m_) were then calculated using the relevant Equation (1).

(1) $$\frac{1}{V}=\left(\frac{1}{\left[S\right]}\right)\left(\frac{K_{m}}{V_{max}}\right)+\frac{1}{V_{max}}$$

Here, V is the enzymatic reaction efficiency (the initial rate of the catalytic reaction at different substrate concentrations). [S] is the concentration of substrate TMB or hydrogen peroxide.

### 2.5. Optimization of Experimental Conditions

Amounts of 150 μL of H_2_O_2_ (100 mM), 250 μL of Ce-MOF (0.05 mg·mL^−1^) and 150 μL of TMB (10 mM) were mixed in a 2 mL centrifuge tube, and 1450 μL of the buffer solution, acetic acid/sodium acetate (pH = 4), was added. The mixture reacted at room temperature (25 °C). During the reaction, Ce-MOF catalyzed TMB to ox-TMB, and the solution was obviously blue. Finally, the UV–vis absorption peak was collected at 652 nm. Similar methods were used to perform optimization experiments with a single variable, and multiple 2 mL centrifugal tubes were prepared. The range of the H_2_O_2_ concentration optimization test was 1–400 mM, and the range of the TMB concentration optimization test was 1–15 mM. The pH optimization test range of the buffer solution of acetic acid/sodium acetate was 2.4–8.2, and the reaction time of the optimized system was 5–50 min.

### 2.6. Selectivity and Anti-Interference Experiment

In order to study the selectivity and anti-interference of the detection system to H_2_S, the same concentration of selected interfering substances was tested under the same detection conditions. Glutamic acid (Glu), glutathione (GSH), cysteine (Cys), anions (NO_2_^−^, S_2_O_4_^2−^, SO_3_^2−^, NO_3_^−^, Br^−^, SO_4_^2−^, CO_3_^2−^) and cations (Na^+^, Ca^2+^, K^+^, Cu^2+^, Mg^2+^, NH_4_^+^) were included. The UV–vis absorbance (the corresponding concentration in the detection system was 192 μM) and fluorescence intensity (the corresponding concentration in the detection system was 320 μM) were measured by adding sodium sulfide and an equal amount of interfering substances, respectively. After the solution completely reacted for 30 min, the solution was placed under the U-3900 ultraviolet spectrophotometer at 652 nm to test its absorbance. All fluorescence spectra were measured using an excitation wavelength of 325 nm. The anti-interference test was conducted to measure the UV–vis absorbance and fluorescence intensity in the same way by adding more than the same amounts of interfering substances in the test system without adding sodium sulfide. After the test was completed, sodium sulfide of equal concentration was added to each tube, and the UV–vis absorbance and fluorescence intensity were determined again [24,25].

### 2.7. Colorimetric–Fluorescence Sensing Analysis

The catalytic activity of the Ce-MOF nanozyme was investigated by using TMB, a typical oxidase substrate, in a buffer solution of acetic acid/sodium acetate (pH = 4). Additionally, 50 mg of sodium sulfide powder was weighed and dissolved in 10 mL of the buffer solution to prepare a 5 mg·mL^−1^ sodium sulfide solution, which was sonicated for future use. A series of 2 mL centrifuge tubes was prepared, each with a total volume of 2 mL of solution. Amounts of 150 μL of H_2_O_2_ (100 mM), 250 μL of Ce-MOF (0.05 mg·mL^−1^) and 150 μL of TMB (10 mM) were added to this mixture, along with varying volumes of acetic acid/sodium acetate buffer (pH = 4) to ensure proper mixing. The final concentration of Ce-MOF in the detection system was 6.25 μg·mL^−1^. The tubes were then incubated at 25 °C for 30 min. Once a noticeable color change was observed, one tube was set aside as a control, and varying volumes of the 5 mg·mL^−1^ sodium sulfide solution, serving as a source of H_2_S, were added to the remaining tubes. The color changes in the solution were visually observed under sunlight and ultraviolet light. The UV–vis absorbance of the solutions was measured at a specific wavelength of 652 nm using a spectrophotometer. Additionally, fluorescence emission spectra were recorded with a fluorescence spectrophotometer, setting the excitation wavelength to 325 nm.

### 2.8. Detection of H_2_S in the Real Samples

The enzyme-labeled strip of 8 tubes was prepared, and 300 μL of buffer solution acetic acid/sodium acetate was added to each tube. The fresh salmon was cut into 25 pieces of similar size and stored in a refrigerator at −20 °C. On days 0–9, four pieces of salmon fillet and solution-containing enzyme-labeled strip were randomly selected every day, sealed in white foam dishes using transparent plastic wrap and kept in a refrigerator at 4 °C. The H_2_S released by the fish body was absorbed by the buffer solution. After 9 days, the test system solution (no buffer solution added) was configured and reacted for 30 min. The solution in the enzyme-labeled strip was dropped into the mixed solution and reacted for 2 min, and colorimetric pictures were taken in a camera obscura. Then, the mixed solution was absorbed into the eight tubes, and an ultraviolet lamp was irradiated to observe the fluorescence change. Shrimp was purchased and frozen at −20 °C. For days 0–5, 5 shrimp and enzyme-labeled strips containing the solution were randomly selected every day, sealed in a white foam plate and kept in a refrigerator at 4 °C. The H_2_S released by the shrimp was absorbed by the buffer solution, and the shrimp was stored for 5 days. Colorimetric and fluorescence changes were observed by the same test method as that used for the salmon.

## 3. Results and Discussion

### 3.1. Characterization

**Figure 1:**
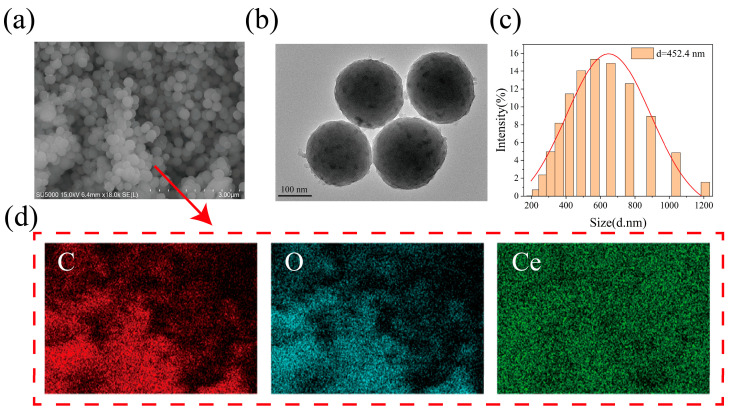
Morphology of Ce-MOF: (**a**) SEM image; (**b**) TEM image; (**c**) DLS analysis; (**d**) EDS images.

Ce-MOF was prepared by the reaction of ammonium cerium nitrate and ligand 2-hydroxyterephthalic acid at 120 °C with DMF as a solvent. The successful preparation of Ce-MOF was confirmed by various characterization techniques [26]. SEM (Figure 1a), TEM and DLS showed the spheroidal morphology characteristic of Ce-MOF, with a size range of about 452.4 nm (Figure 1c). Higher resolution images by TEM showed uniform-sized nanoparticles of Ce-MOF (Figure 1b), which was further verified by dynamic light scattering results. X-ray energy dispersive spectroscopy (EDS) showed that Ce-MOF contained Ce, C and O elements, and all elements were evenly dispersed in the Ce-MOF nanozyme structure (Figure 1d).

**Figure 2:**
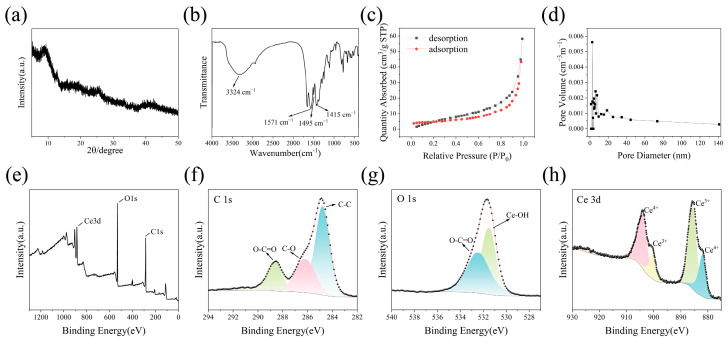
Characterization of Ce-MOF: (**a**) XRD spectra; (**b**) FT-IR spectra; (**c**) Nitrogen adsorption–desorption isotherm; (**d**) BJH pore diameter; (**e**) XPS spectra; (**f**) C 1s spectra; (**g**) O 1s spectra; (**h**) Ce 3d spectra.

The XRD pattern of Ce-MOF displayed three diffraction peaks centered at 8.9°, 25.6° and 40.7° (Figure 2a), which aligned well with the spectra synthesized by the predecessors [27], and the successful synthesis of Ce-MOF was confirmed. In the FTIR spectrum (Figure 2b), extensive asymmetric stretching vibrations at 3324 cm^−1^ indicated abundant hydroxyl groups (O-H) on the surface of Ce-MOF. The peak at 1415 cm^−1^ corresponded to the symmetric stretching of the carboxylic acid group (O=C–O), and asymmetric stretching vibrations were observed at 1571 cm^−1^. Additionally, absorption at 1495 cm^−1^ suggested the presence of asymmetric C=C tensile vibrations in the benzene ring [28]. These results verify the successful coordination of ammonium cerium nitrate with 2-hydroxyterephthalic acid [29]. The absolute surface value for Ce-MOF was 16.353 m^2^/g. According to Figure 2c, the adsorption isotherm of Ce-MOF exhibited an inflection point within the low-pressure region. The adsorption and desorption curve did not overlap in the high-pressure region, and there was a hysteresis loop. According to the classification of the adsorption isotherms, characteristics of type II and type IV were seen, indicating that they are mesoporous materials [30]. According to Figure 2d, the size diagram of the aperture had an average size of 2.52 nm. This can prove that Ce-MOF is a mesoporous material.

XPS spectra were analyzed to elucidate the surface composition of Ce-MOF, particularly focusing on the oxidation states of cerium (Ce). The comprehensive XPS spectrum revealed Ce 3d, O 1s and C 1s signals, consistent with the elemental mapping (Figure 2e). High-resolution XPS spectra of Ce 3d indicated the coexistence of Ce^4+^ and Ce^3+^ on the surface of Ce-MOF (Figure 2h). Herein, 886.28 and 900.48 eV belong to the valance state of 3^+^, and 881.58 and 904.48 eV denote the 4^+^ state for Ce [31]. Moreover, the high-resolution C 1s spectrum displayed peaks at 286.18, 284.78 and 288.58 eV, corresponding to C–O, C–C and O–C=O bonds (Figure 2f), and the O 1s spectrum showed peaks at 532.48 and 531.48 eV for O–C=O and Ce–OH, confirming the chemical environment of the surface (Figure 2g) [22].

### 3.2. Peroxidase Mimetic Activity Analysis of Ce-MOF

The peroxidase-like activity of Ce-MOF was evaluated using TMB as the substrate. Experimental parameters, including pH, TMB concentration, H_2_O_2_ concentration and reaction time, were optimized to maximize peroxidase activity. The optimal conditions were determined to be a pH of 4, a TMB concentration of 10 mM and an H_2_O_2_ concentration of 100 mM. After a reaction time of 30 min, the color change in the solution was readily observable to the naked eye. Thus, the optimal reaction duration was established at 30 min under balancing detection efficacy and visibility.

**Figure 3:**
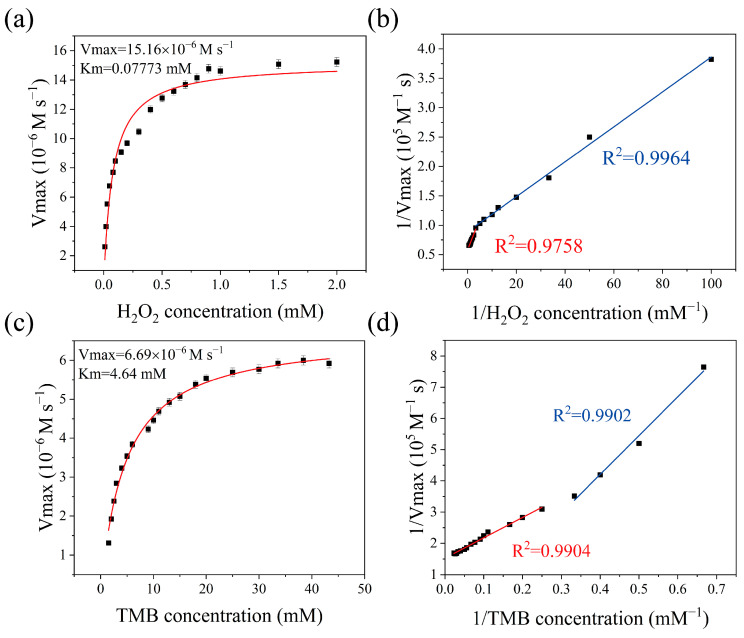
Steady-state kinetic analysis: (**a**,**b**) Kinetic study for the substrate of H_2_O_2_ of Ce-MOF based on the Michaelis–Menten equation; (**c**,**d**) Kinetic study for the substrate of TMB of Ce-MOF based on the Michaelis–Menten equation.

A steady-state kinetic analysis was conducted to assess the peroxidase mimetic activity of Ce-MOF. As the TMB concentration increased from 1.5 mM to 43.26 mM and as the H_2_O_2_ concentration rose from 0.01 mM to 2 mM, the reaction rate of Ce-MOF peroxidase steadily increased. This response exhibited typical Michaelis–Menten kinetics, indicating that substrate catalysis adhered to the characteristics of this model. The values of K_m_ and V_max_ were determined through fitting the Lineweaver–Burk plot (Figure 3). The K_m_ value for Ce-MOF with H_2_O_2_ was 0.07773 mM, which reflects the affinity between the enzyme and its substrate. A lower K_m_ value indicates a stronger affinity between Ce-MOF and its substrate compared to natural horseradish peroxidase (HRP) and other reported nanozymes. Ce-MOF exhibited comparable or superior affinity and catalytic efficiency for H_2_O_2_, suggesting enhanced catalytic activity, making it suitable for use in subsequent colorimetric assays. At the same time, the maximum reaction rates of the Ce-MOF substrates, H_2_O_2_ and TMB, were 15.16 × 10^−6^ Ms^−1^ and 6.69 × 10^−6^ Ms^−1^, respectively, which were also higher than the maximum reaction rates of some nanozymes toward H_2_O_2_ substrates (Table 1), indicating that Ce-MOF has excellent and higher catalytic activity. It also exhibited strong peroxidase-mimicking activity [32].

### 3.3. Colorimetric Characteristic Analysis of Ce-MOF

**Figure 4:**
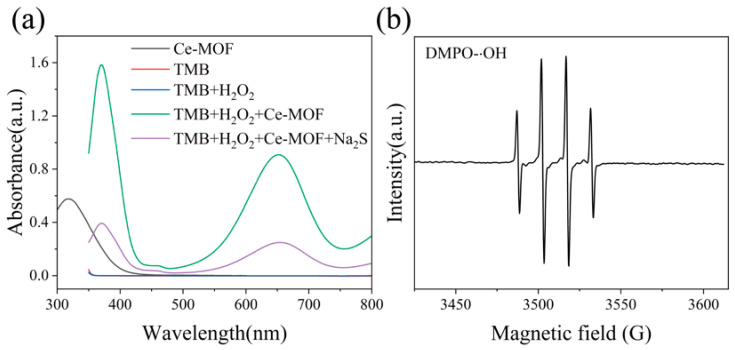
Colorimetric characteristic analysis: (**a**) Feasibility of colorimetric detection of dual-mode sensor; (**b**) EPR spectrum of DMPO- OH.

The feasibility analysis of the dual-mode colorimetric–fluorescence sensor was carried out according to a previous method [39]. As shown in Figure 4a, TMB alone (purple line) and TMB combined with H_2_O_2_ (red line) exhibited no significant UV–vis absorption peak at 652 nm. However, upon the addition of Ce-MOF (blue line), the UV–vis absorption spectrum displayed a pronounced peak at 652 nm. This peak was notably diminished when the mixture was treated with a 5 mg·mL^−1^ sodium sulfide solution, indicating a significant reduction in the concentration of Ce-MOF following the absorption of H_2_S. This observation could also suggest that the catalytic activity of the Ce-MOF nanozyme was substantially decreased after the addition of H_2_S, and it implies that the presence of H_2_S might inhibit the Ce-MOF nanozyme’s catalysis in the TMB oxidation reaction [32].

In order to identify the active free radicals produced during Ce-MOF catalysis, the EPR technique was used to directly detect reactive oxygen species (ROS) using 5,5-dimethyl-1-pyrroline N-oxide (DMPO) as a trapping probe. It could be observed that ·OH (hydroxyl radical) radiated in the Ce-MOF solution for 20 min in a four-wire spectrum of 1:2:2:1, which further confirms that the hydroxyl radical was the main intermediate, and ·OH was produced in the system, from which it can be inferred that ·OH was the main ROS in the Ce-MOF system (Figure 4b) [40]. It was verified that the catalytic pathway of the solution involved generating a hydroxyl radical, oxidizing TMB to oxTMB and changing the solution from colorless to blue [41].

**Figure 5:**
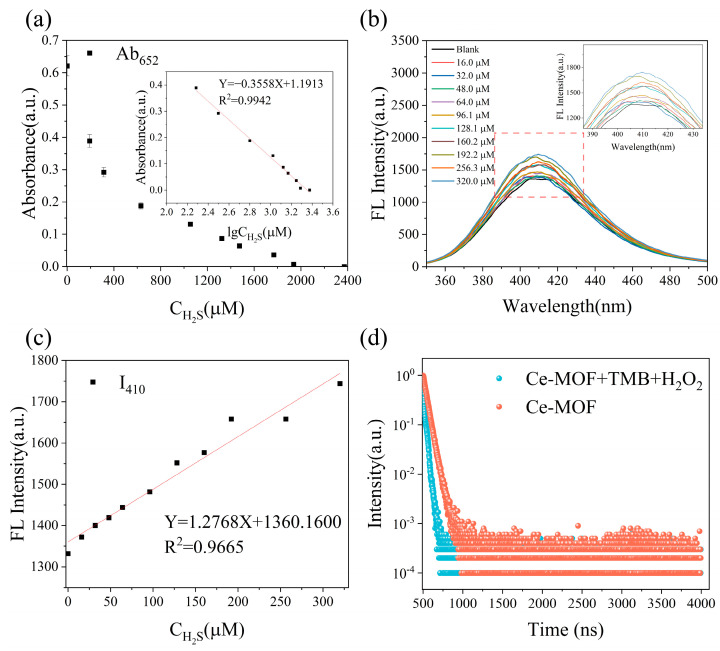
Dual-mode colorimetric–fluorescence sensor responds to different H_2_S concentrations: (**a**) Linear relationship between UV–vis absorption and different H_2_S concentrations; (**b**) Fluorescence spectrum of different H_2_S concentrations under excitation of 325 nm; (**c**) Linear relationship between fluorescence spectrum and different H_2_S concentrations; (**d**) Fluorescence lifetime of Ce-MOF.

Ten 2 mL centrifuge tubes were prepared, and 150 μL of H_2_O_2_ (100 mM), 250 μL of Ce-MOF (0.05 mg·mL^−1^) and 150 μL of TMB (10 mM) were added, respectively, and mixed with different volumes of the buffer solution of acetic acid/sodium acetate for 30 min, making sure that the solution was blue after the full reaction. After the reaction, 0.0, 6.0, 9.8, 19.7, 32.9, 41.3, 46.0, 55.2, 60.5 and 74.0 μL sodium sulfide solutions were added to ten 2 mL centrifuge tubes, and the total volume of the mixed solution in each centrifuge tube was fixed to be 2 mL. The concentration of H_2_S in the system was 0.000, 0.192, 0.314, 0.631, 1.054, 1.323, 1.474, 1.768, 1.938 and 2.371 mM, respectively. After 2 min, the absorption of the series of concentration gradient solutions at 652 nm was determined, and the UV–vis absorption spectrum was obtained. As the concentration of sodium sulfide increased, the characteristic peak of oxTMB gradually decreased, and it could be seen that the UV–vis absorption of the sensor had a good correlation with the concentration of Na_2_S (0–2300 μM) (Figure 5a). By fitting this value to the Na_2_S concentration, the fitted equation was Y = −0.3558X + 1.1913 (R^2^ = 0.9942) with a limit of detection (LOD) of 0.262 µM (Figure 5a inset). The limit of detection was calculated by Equation (2), where σ is the standard deviation of the blank and k is the slope of the linear fitting equation.

(2) $$\mathrm{LOD}=\frac{3\sigma }{k}$$

The sensor colorimetric response had a detection range of 200–2300 µM. As the concentration of H_2_S in the solution increased, the color transitioned from blue to colorless, demonstrating the inhibitory effect of H_2_S on the catalytic activity of Ce-MOF. This effect could be attributed to several mechanisms, including strong coordination between H_2_S and the active sites of Ce-MOF, a reduction in the formation of intermediate hydroxyl radicals and a decrease in the levels of peroxide or oxTMB.

### 3.4. Analysis of Fluorescence Properties of Ce-MOF

The excitation wavelength of Ce-MOF was determined by the UV–vis absorption curve. It can be seen from Figure 4a that, when only Ce-MOF existed in solution, the wavelength corresponding to the UV–vis absorption peak appeared at 325 ± 5 nm. Therefore, 325 nm was set as the fluorescence emission of the excitation wavelength determination test system. The peak fluorescence emission occurred at 410 ± 5 nm. Eleven 2 mL centrifuge tubes were prepared and mixed with 150 μL of H_2_O_2_ (100 mM), 250 μL of Ce-MOF (0.05 mg·mL^−1^), 150 μL of TMB (10 mM) and different volumes of the buffer solution acetic acid/sodium acetate for 30 min, making sure that the solution was blue after the full reaction. After the reaction, 0.0, 0.5, 1.0, 1.5, 2.0, 3.0, 4.0, 5.0, 6.0, 8.0 and 10.0 μL sodium sulfide solutions that had turned were added to eleven 2 mL centrifugal tubes, and the total volume of the mixed solution in each centrifugal tube was also fixed to 2 mL. The concentration of H_2_S in the system was 0.0, 16.0, 32.0, 48.0, 64.0, 96.1, 128.1, 160.2, 192.2, 256.3 and 320.0 μM, respectively. After the reaction for 2 min, the fluorescence intensity of this series of concentration gradient solutions was determined at an excitation wavelength of 325 nm, and the fluorescence spectrum, as shown in Figure 5b, was obtained.

In order to further investigate the fluorescence response of Ce-MOF to H_2_S, the emission spectra of Ce-MOF treated with different concentrations of H_2_S (0–320 μM) in the buffer solution of acetic acid/sodium acetate were recorded. As shown in Figure 5b, the fluorescence intensity of Ce-MOF at 410 nm gradually increased with the increase in the concentration of Na_2_S in the H_2_S source. A rate-based fluorescence method was used to evaluate the detection ability of Ce-MOF. As shown in Figure 5c, the fluorescence intensity of the sensor I_410_ had a good correlation with the concentration of Na_2_S (0–320 μM). The fitting equation is as follows: Y = 1.2768X + 1360.1600 (R^2^ = 0.9665). More importantly, under the irradiation of 365 nm ultraviolet light, the color of the detection liquid showed a significant change from no obvious fluorescence to obvious bright blue fluorescence, which could realize the visual detection of the sensor. The Ce-MOF sensor had a detection range of 0–320 μM for H_2_S. With a limit of detection of 0.156 μM, the detection of H_2_S by Ce-MOF was more sensitive than previously reported. The colorimetric detection method was directly visible to the naked eye in sunlight and was simple and portable, so we fabricated a colorimetric sensing method. According to Table 2, it can be seen that the colorimetric limit of detection of our sensor was lower than that of other colorimetric sensors but still higher than that of most fluorescence sensors. Therefore, we also established a more precise fluorescence detection method, and a dual-mode colorimetric–fluorescent sensor was made in our work. By comparing the LOD values of the sensors studied by previous researchers, it can be found that our Ce-MOF material had a low LOD among a variety of sensors using colorimetric, fluorescent or dual-mode colorimetric–fluorescence methods, indicating its high sensitivity.

It can be seen from the ultraviolet curve and fluorescence spectrum that the fluorescence emission wavelength of the sensor ranged from 350 nm–500 nm, and the blue line (TMB + H_2_O_2_ + Ce-MOF) and green line (TMB + H_2_O_2_ + Ce-MOF + Na_2_S) in the UV–vis absorption spectrum peaked at around 350 nm–450 nm. Fluorescence lifetimes were used to explore the mechanisms underlying the establishment of the dual-mode Ce-MOF nanozyme sensor. The overlap of the fluorescence emission spectrum of Ce-MOF (energy donor) and the UV–vis absorption spectrum of oxTMB (energy acceptor) shown in Figure 4a provide the conditions of FRET. As shown in Figure 5d, the fluorescence lifetime of Ce-MOF was 24 ns, which changed to 16 ns after oxTMB was added. The change in the fluorescence lifetime indicated that the enhanced fluorescence between Ce-MOF and oxTMB was based on the shortened fluorescence lifetime of Ce-MOF as an energy donor by FRET [42]. In addition, with the increase in the concentration of Na_2_S, the oxTMB in the solution was reduced to TMB, the oxTMB decreased, and the fluorescence emission intensity of the detection system at 410 nm gradually increased, indicating that the FRET effect was enhanced [43,44].

**Table 2:** Comparison of other sensors for detecting H_2_S.

Sensors	Types	LOD	Ref.
CR-DNP	Fluorescence	0.4 μM	[45]
Pb(btc)-1	Colorimetric	110 μM	[46]
P1	Fluorescence	0.66 μM	[47]
Probe L	Fluorescence	0.372 μM	[5]
Microplate cover-based colorimetric assay	Colorimetric	1.48 μM	[48]
Eu(tdl)_2_abp	Fluorescence	0.64 μM	[49]
GG-AgNPs	Colorimetric	0.81 μM	[50]
Ce-MOF	Colorimetric	0.262 μM	This work
	Fluorescence	0.156 μM	

### 3.5. Selectivity and Anti-Interference

The evaluation of selectivity and anti-interference performance are key factors in the analysis of accuracy. To evaluate the specific perception of H_2_S by Ce-MOF, Glu, GSH and Cys, various potential interfering substances such as anions (NO_2_^−^, S_2_O_4_^2−^, SO_3_^2−^, NO_3_^−^, Br^−^, SO_4_^2−^, CO_3_^2−^) and cations (Na^+^, Ca^2+^, K^+^, Cu^2+^, Mg^2+^, NH^4+^) were added into the detection system in equal amounts under the same concentration test conditions. All UV–vis absorbance and fluorescence spectra were recorded.

**Figure 6:**
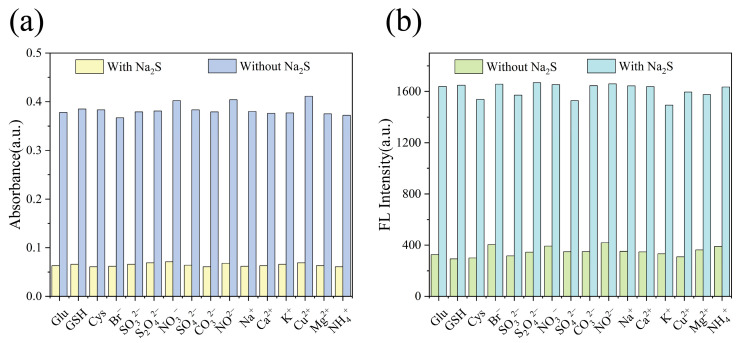
Selectivity and anti-interference of Ce-MOF: (**a**) UV–vis absorption spectrum from the responses of Ce-MOF in the presence of H_2_S or coexistence with other species; (**b**) Fluorescence intensity from the responses of Ce-MOF in the presence of H_2_S or coexistence with other species.

As can be clearly seen in Figure 6a,b, the presence of these interfering substances did not cause a significant change in the color of the reaction solution. In contrast, the peak UV–vis absorption was significantly reduced, and the fluorescence intensity was significantly enhanced after the introduction of H_2_S. In the selective test, it is worth noting that sulfite, thiosulfate and cysteine, which are very similar to H_2_S, had little effect on the Ce-MOF nanozyme, and their UV–vis absorbance ratio and fluorescence intensity ratio were not obvious compared with H_2_S. These results show that the Ce-MOF-nanozyme-based H_2_S detection method had excellent selectivity and anti-interference capability, and the established method had significant selectivity and could specifically distinguish and detect H_2_S despite the presence of potential interfering compounds.

### 3.6. Detection of H_2_S e in Real Samples

**Figure 7:**
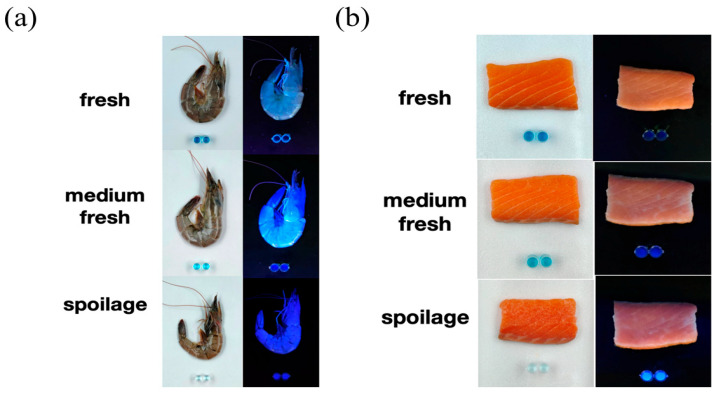
Application of Ce-MOF in real samples: (**a**) H_2_S detection in shrimp; (**b**) H_2_S detection in salmon.

Monitoring H_2_S concentrations is used as a fast, sensitive, non-destructive and stable method to evaluate the freshness of aquatic products. Fresh salmon and shrimp were selected as the real detected objects, and the colorimetric and fluorescence changes in Ce-MOF sensors were monitored at 4 °C for 9 days of salmon deterioration and 5 days of shrimp deterioration (Figure 7a). The salmon stored at 4 °C were selected on days 0, 4 and 9, and the shrimp samples were picked on days 0, 2 and 4 (Figure 7b). The buffer solution of acetic acid/sodium acetate on different days reacted with the mixed solution of the test system for 2 min, and the color changes in the solution were observed under daylight and ultraviolet light, respectively. It was observed that the colorimetry of the sensor gradually changed from blue to colorless, and the fluorescence of the sensor gradually increased, which further confirmed that the sensor had a significant response to different H_2_S concentrations generated by decomposition during the degradation of aquatic products [51].

## 4. Conclusions

In summary, this work proposed and fabricated a novel nanozyme dual-mode sensor and established a colorimetric–fluorescence method for the real-time visual determination of H_2_S. This was achieved by utilizing Ce-MOF as a peroxidase mimetic enzyme and a dual-mode probe. The Ce-MOF nanozyme exhibited a rapid response (<5 min), high selectivity and exceptional sensitivity (LOD = 0.262 μM in colorimetric method; LOD = 0.156 μM in fluorescence method). The sensor comprised Ce-MOF, 3,3’,5,5’-tetramethylbenzidine (TMB) and H_2_O_2_. Ce-MOF acted as a catalyst for hydrogen peroxide decomposition, generating ·OH that oxidized TMB into oxTMB. Additionally, energy transferred from Ce-MOF to TMB through FRET. As the concentration of H_2_S increased in the solution, the FRET effect strengthened, leading to enhanced fluorescence signals with gradient intensity. Furthermore, this sensor was successfully applied to detect H_2_S contents in salmon and shrimp samples, which allowed us to accurately monitor their freshness levels, ranging from fresh to medium fresh to spoiled states. In summary, the improved nanozyme dual-mode sensor not only enabled more precise detection of H_2_S but also demonstrated excellent visualization performance when analyzing real samples such as food products. 

## Figures and Tables

**Table 1:** Comparison of kinetic parameters of H_2_O_2_.

Nanozymes	*K*_m_ (mM)	*V*_max_ (10^−6^ Ms^−1^)	Ref.
Fe_3_O_4_NPs	154	5.7333	[33]
MOF-808	1.06	0.0139	[34]
Au-Ni/g-C_3_N_4_	0.16	0.0234	[35]
PtCu	0.37	0.0114	[36]
Ag-MoS_2_	2.29	0.0875	[37]
HRP	3.7	0.0870	[38]
Ce-MOF	0.07773	15.16	This work

## Data Availability

All data analyzed in this study are included in the published article.
